# *Pseudoalteromonas haloplanktis* TAC125 produces 4-hydroxybenzoic acid that induces pyroptosis in human A459 lung adenocarcinoma cells

**DOI:** 10.1038/s41598-018-19536-2

**Published:** 2018-01-19

**Authors:** Filomena Sannino, Clementina Sansone, Christian Galasso, Sara Kildgaard, Pietro Tedesco, Renato Fani, Gennaro Marino, Donatella de Pascale, Adrianna Ianora, Ermenegilda Parrilli, Thomas Ostenfeld Larsen, Giovanna Romano, Maria Luisa Tutino

**Affiliations:** 1 Department of Chemical Sciences, University of Naples “Federico II”, Complesso Universitario Monte S. Angelo, Via Cintia, Naples, 80126 Italy; 2 Integrative Marine Ecology Department, Stazione Zoologica Anton Dohrn, Villa Comunale, Naples, 80121 Italy; 3 Department of Biotechnology and Biomedicine, Søltofts Plads, Building 221, Technical University of Denmark, DK-2800 Kgs. Lyngby, Denmark; 4 Institute of Protein Biochemistry, CNR, Via Pietro Castellino 111, Naples, 80131 Italy; 5 Department of Biology, LEMM, Laboratory of Microbial and Molecular Evolution Florence, University of Florence, I-50019 Sesto Fiorentino (FI), Italy; 6 University Suor Orsola Benincasa, Via Santa Caterina da Siena, 32, Naples, 80132 Italy

## Abstract

In order to exploit the rich reservoir of marine cold-adapted bacteria as a source of bioactive metabolites, ethyl acetate crude extracts of thirteen polar marine bacteria were tested for their antiproliferative activity on A549 lung epithelial cancer cells. The crude extract from *Pseudoalteromonas haloplanktis* TAC125 was the most active in inhibiting cell proliferation. Extensive bioassay-guided purification and mass spectrometric characterization allowed the identification of 4-hydroxybenzoic acid (4-HBA) as the molecule responsible for this bioactivity. We further demonstrate that 4-HBA inhibits A549 cancer cell proliferation with an IC_50_ value ≤ 1 μg ml^−1^, and that the effect is specific, since the other two HBA isomers (i.e. 2-HBA and 3-HBA) were unable to inhibit cell proliferation. The effect of 4-HBA is also selective since treatment of normal lung epithelial cells (WI-38) with 4-HBA did not affect cell viability. Finally, we show that 4-HBA is able to activate, at the gene and protein levels, a specific cell death signaling pathway named pyroptosis. Accordingly, the treatment of A549 cells with 4-HBA induces the transcription of (amongst others) caspase-1, IL1β, and IL18 encoding genes. Studies needed for the elucidation of mode of action of 4-HBA will be instrumental in depicting novel details of pyroptosis.

## Introduction

Lung cancer is an extremely important health concern that affects millions of people worldwide^1,2^, and any progress leading to improvement of cancer survival rates is a global priority. Patients with lung cancer generally have a poor prognosis with a 5-year survival^2^. Traditional cancer chemotherapy has mainly been based on the use of highly cytotoxic drugs that non-specifically target all dividing cells and may therefore only result in a modest improvement in patients that become immunosuppressed as chemotherapeutics kill all proliferating cells including monocytes and lymphocytes. For this reason, a new trend in anticancer research has arisen focusing on the discovery of new natural drugs that induce specific programmed cell death mediated by immunogenic signals. A recently discovered form of immunogenic cell death is represented by pyroptosis. This pathway differs from that of apoptosis as it is uniquely mediated by caspase-1 (CASP1) activation, which in turn triggers the formation of an “inflammasome”, a cytosolic complex with inflammatory features^3^ linked to interleukin 1β (IL1β) release for immune cell recruitment.

Many of the anticancer drugs used in clinical practice today are natural products or derivatives thereof^4^ and the continued and systematic exploration of natural sources, such as marine microbiota, is expected to lead to the discovery of different and unforeseen compounds with interesting biological activities, including anticancer activity^5^. Marine bacteria have proven to be a unique and promising source of biologically active natural products^6^. The production of anticancer drugs by microorganisms can be advantageous in comparison to other natural sources, such as plants, due to i) the possibility of genetically engineering microbes and ii) their higher production rates^7^.

Amongst marine bacteria, cold-adapted microorganisms represent an untapped reservoir of biodiversity endowed with an interesting chemical repertoire. It has been already shown that cold-adapted bacteria produce valuable bioactive secondary metabolites, such as anti-biofilm molecules^8–10^, antimicrobials^11,12^ and compounds displaying various other pharmaceutically-relevant activities^13^. In this context, polar marine bacteria could likely be a potential source of new molecules with antiproliferative activity.

In the present study we screened ethyl acetate extracts of thirteen different cultivable cold-adapted bacteria on A549 cells, a lung adenocarcinoma cell line, which represents a suitable model for the study of Non Small Cancer Lung Cells having typical characteristics in terms of proliferation index and malignancy^14^. We demonstrate that *Pseudoalteromonas haloplanktis* TAC125^15,16^ (*P. haloplanktis* TAC125) is able to produce an antiproliferative agent. In particular, this bacterium produces 4-hydroxybenzoic acid that specifically activates pyroptosis in A549 cells without affecting viability in normal cells.

## Results

### Screening for antiproliferative activity of polar bacteria ethyl acetate extracts, and production conditions optimization

Ethyl acetate crude extracts of thirteen bacterial strains (Table S1) were tested for their antiproliferative activity using the MTT assay on lung adenocarcinoma A549^14^ cells. The ethyl acetate extract of uninoculated GG medium was used as a negative control. A549 tumor cells were treated with different concentrations (1, 10 and 100 μg ml^−1^) of the total extracts for 24 hours (data not shown) and 48 hours, and compared with cells treated with the negative control extract. The highest concentration tested (100 μg ml^−1^) induced a decrease in the percentage of viable cells for most of the extracts (Figure S1). Interestingly, the crude extract of *P. haloplanktis* TAC125 inhibited cell viability in a dose dependent manner, with a calculated half Inhibition Concentration (IC_50_) of about 30 μg ml^−1^. Based on these results, we focused on *P. haloplanktis* TAC125 as a potential source of antiproliferative compounds.

To test whether the composition of growth medium affected the production of antiproliferative compound(s), the Antarctic bacterium was grown in different media: a rich medium (TYP)^17^; a synthetic medium (GG)^16^; and a medium containing alternatively L-glutamate or D-gluconate as source of carbon and nitrogen. Extract of *P. haloplanktis* TAC125 grown in GG medium displayed the highest antiproliferative activity on A549 cells (data not shown).

To increase production of antiproliferative compound(s), *P. haloplanktis* TAC125 was grown in GG medium in an automatic bioreactor. The cell extract of *P. haloplanktis* TAC125 grown in the bioreactor displayed an IC_50_ value of about 1 μg ml^−1^, indicating a clear enhancement in production yields under this growth condition (Figure S2).

### Bioactivity guided purification and identification of the antiproliferative compound from *Pseudoalteromonas haloplanktis* TAC125

**Figure 1:**
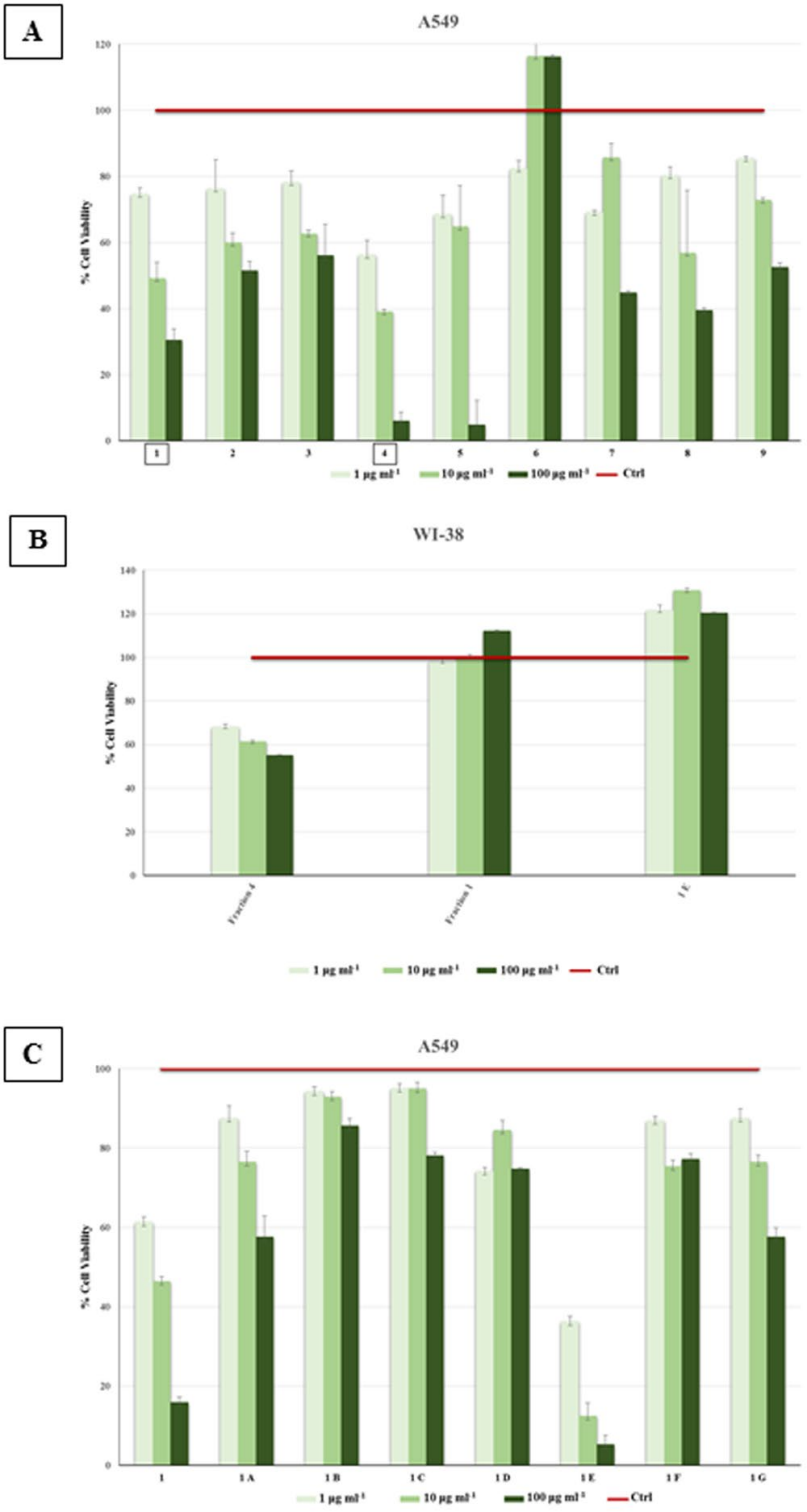
Panel (A): Cell viability of lung adenocarcinoma cells (A549) treated for 48 hours with three different concentrations (1, 10 and 100 µg ml^−1^) of 9 fractions of *Pseudoalteromonas haloplanktis* TAC125 obtained on a C_18_ column by a Isolera One purification system. Panel (B): Cell viability of lung normal fibroblast cells (WI-38) treated for 48 hours with three different concentrations (1, 10 and 100 µg ml^−1^) of three samples: Fraction 4 and Fraction 1 from fractionation on a C18 column by Isolera One system; 1E from following purification step by HPLC of the Fraction 1. Panel (C): Cell viability of lung adenocarcinoma cells (A549) treated for 48 hours with three different concentrations (1, 10 and 100 µg ml^−1^) of 7 fractions (from 1A to 1G) of *Pseudoalteromonas haloplanktis* TAC125 obtained from Fraction 1 on a semipreparative HPLC. In all the above experiments, red bar represents untreated cells (control). Three independent assays were performed in triplicate; viability data are shown as mean ± S.D.

Pre-fractionation of the *P. haloplanktis* TAC125 extract was achieved on a reversed phase column, and fractions were tested by the MTT assay on A549 lung cancer cells (Fig. 1, panel A). Fractions 1 and 4 were shown to inhibit A549 cells in a dose dependent manner and both fractions displayed IC_50_ values of about 10 μg ml^−1^. Fraction 4 was discarded as it was also shown to exert a high cytotoxicity towards the non transformed lung epithelial WI-38 cells (Fig. 1, panel B).

The bioactive fraction 1 was subjected to a further purification step and the resulting fractions were tested for antiproliferative activity with the MTT assay on A549 cells (Fig. 1, panel C). Fraction 1E was identified as the most active with an IC_50_ value of about 0,8 μg ml^−1^ and very interestingly this fraction displayed no toxic effect towards the WI-38 normal cell line (Fig. 1, panel B).

The active fraction 1E was analysed by Ultra-high performance liquid chromatography-diode array detection-high-resolution mass spectrometry (UHPLC-DAD-HRMS) with tandem MS/HRMS fragmentation that revealed a single compound which was tentatively identified as 4-hydroxybenzoic acid (4-HBA) from a search in a comprehensive in-house standard collection of microbial metabolites^18^.

**Figure 2:**
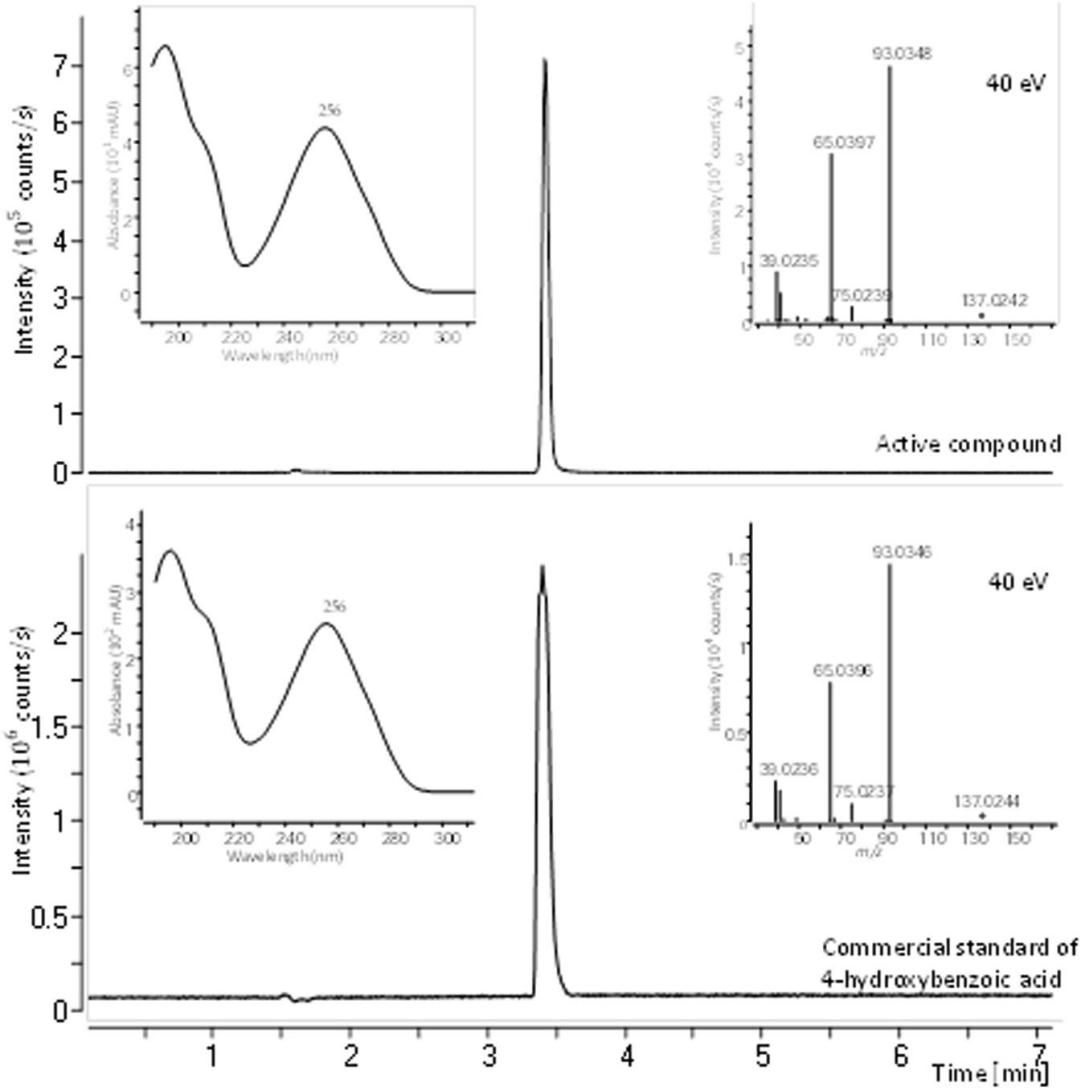
ESI ^−^ extracted ion chromatograms, UV spectra and MS/HRMS spectra at 40 eV for the single active compound (1E) and commercial standard of 4-hydroxybenzoic acid (4-HBA).

The identification of 4-HBA was verified by comparison of retention time, monoisotopic mass for the pseudomolecular ion [M-H]^−^, MS/HRMS spectra (10 eV, 20 eV and 40 eV) from negative electrospray ionization (ESI) mode and UV spectrum with a commercially available standard (Fig. 2).

A comparable cytotoxic effect was displayed for the isolated pure compound (1E) and the commercial standard of 4-HBA when tested in the same experiment using the MTT assay on A549 cells (Figure S3). To investigate the structural features essential for this antiproliferative activity, the effect of the hydroxybenzoic acid structural isomers (2-, 3-, and 4-HBA) were analyzed. The results of the MTT assay on A549 cells indicated that 4-HBA was the most active isomer, with an IC_50_ ≤ 1 μg ml^−1^ (Figure S4).

### Mechanism of action for 4-hydroxybenzoic acid

**Figure 3:**
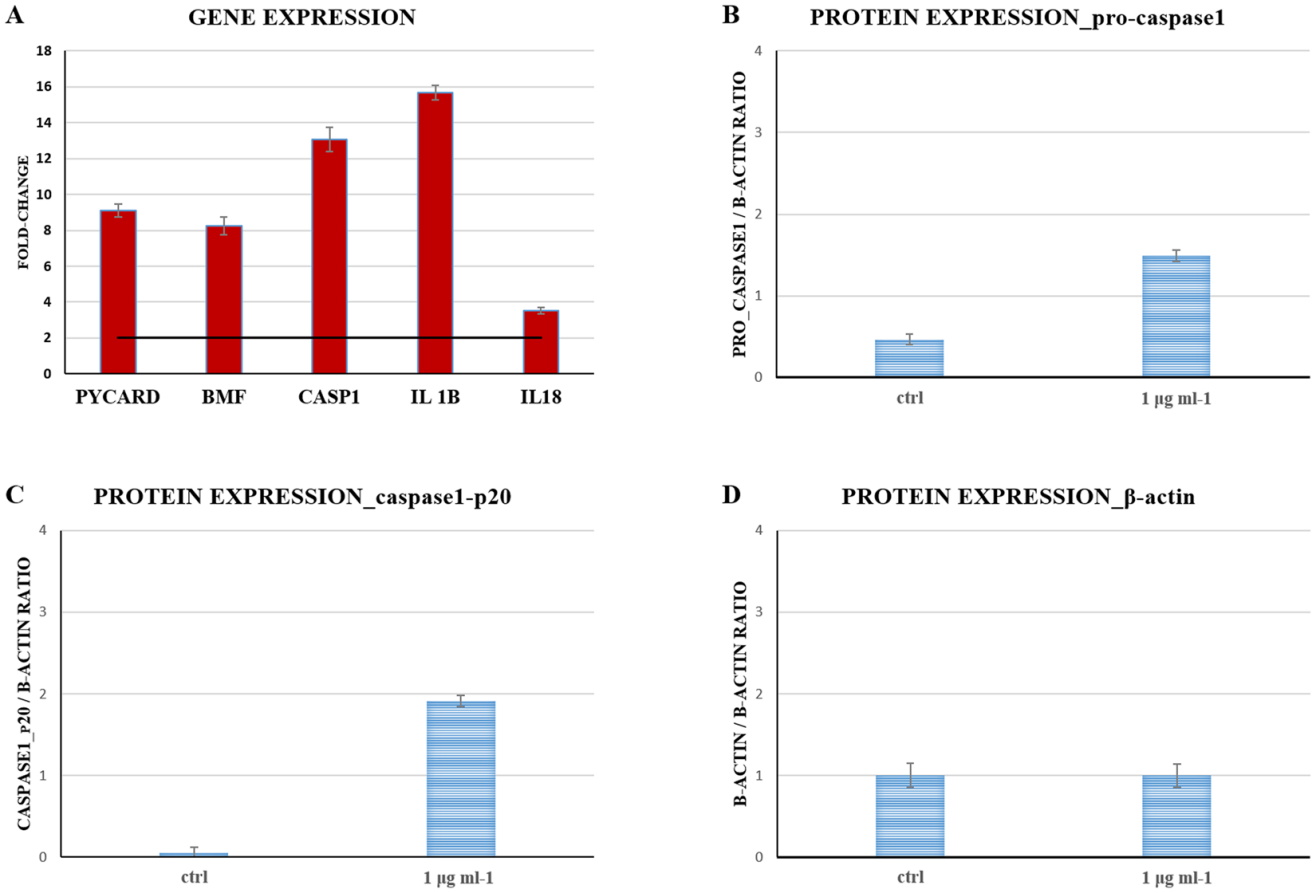
Histograms showing the effects of 4-HBA (1 µg ml^−1^, IC_50_ concentration) on target genes expression and proteins levels in A549 lung adenocarcinoma cells. Panel (A): Gene expression analysis after 2 hours of treatment with the compound; three independent assays were performed in triplicate and the data are expressed as mean ± S.D. Expression values greater or lower than a two-fold difference with respect to the controls were considered significant (black bar). Panels (B, C and D): Histograms show the effects after 24 hours of 4-HBA treatment (1 µg ml^−1^, IC_50_ concentration) on expression levels of target proteins in A549 lung adenocarcinoma cells. (**B)** Pro_Caspase1; (**C)** Caspase1_p20 cleavage fragment; (**D**) control protein, β-Actin. Three independent assays were performed in triplicate; data are shown as mean ± S.D.

The specific molecular pathway activated by 4-HBA was defined through a PCR array experiment aimed to evaluate changes in expression of the main genes involved in the most common signaling pathways of cell death^19^ (Table 1). A549 cells were treated with 1 µg ml^−1^ of 4-HBA (IC_50_ concentration) and after 2 hours of incubation, cells were recovered and subjected to a real time qPCR analysis. Only two-fold difference in expression values with respect to the control (untreated cells) were used to identify up- and/or down-regulated genes, respectively (Table 1). We found that the expression of the following key genes involved in pyroptotic cell death signalling^20^ were all up-regulated including the Caspase Recruitment Domain-Containing Protein 5 (PYCARD), Bcl2 Modifying Factor (BMF), Caspase-1 (CASP1), Interleukin-1β (IL1β) and Interleukin-18 (IL18) (Fig. 3, panel A, Table 1).

**Table 1:** Transcriptional modulation of a subset of genes involved in human death cell signalling pathways in 4-HBA treated A549 cells. Gene transcription is considered unaffected by compound treatment if fold regulation is in the range ± 2.0.

Unigene	Refseq	Symbol	Description	Fold Regulation	St. deviation
Genes up-regulated by 4-HBA treatment					
Hs.249227	NM_130463	PYCARD	Caspase Recruitment Domain-Containing Protein 5	9.1022	0.009952
Hs.591104	NM_033503	BMF	Bcl2 modifying factor	8.2385	0.009952
Hs.2490	NM_033292	CASP1	Caspase 1, apoptosis-related cysteine peptidase (interleukin 1, beta, convertase)	13.0597	1.382987
Hs.743398	NM_213607	IL1B	Interleukin-1 beta	15.6858	5.424528
Hs.472860	NM_001250	IL18	Interleukin-18	3.523	3.141189
Hs.32949	NM_005218	DEFB1	Defensin, beta 1	16.3404	4.718064
Hs.656958	NM_002647	PIK3C3	Phosphoinositide-3-kinase, class 3	2.0217	0.291856
Hs.356076	NM_001167	XIAP	X-linked inhibitor of apoptosis	2.0656	0.551179
Genes down-regulated by 4-HBA treatment					
Hs.592244	NM_000074	CD40LG	CD40 ligand	−12.6362	0.009952
Hs.744830	NM_000125	ESR1	Estrogen receptor 1	−3.7381	0.545221
Hs.2007	NM_000639	FASLG	Fas ligand (TNF superfamily, member 6)	−13.6362	0.009952
Hs.87236	NM_012188	FOXI1	Forkhead box I1	−13.2454	0.09952
Hs.518450	NM_002111	HTT	Huntingtin	−2.13	0.109509
Hs.856	NM_000619	IFNG	Interferon, gamma	−45.0004	2.581419
Hs.160562	NM_000618	IGF1	Insulin-like growth factor 1 (somatomedin C)	−3.8688	1.644252
Hs.700350	NM_000207	INS	Insulin	−6.5181	1.580485
Hs.519680	NM_001145805	IRGM	Immunity-related GTPase family, M	−12.6362	0.009952
Hs.592068	NM_020655	JPH3	Junctophilin 3	−4.7242	0.148375
Hs.484111	NM_014592	KCNIP1	Kv channel interacting protein 1	−14.7747	0.309055
Hs.643440	NM_002361	MAG	Myelin associated glycoprotein	−147.3426	5.001374
Hs.553833	NM_001004467	OR10J3	Olfactory receptor, family 10, subfamily J, member 3	−15.6362	0.009952
Hs.632469	NM_020387	RAB25	RAB25, member RAS oncogene family	−10.6362	0.009952
Hs.442337	NM_176823	S100A7A	S100 calcium binding protein A7A	−170.5556	5.299868
Hs.202676	NM_014258	SYCP2	Synaptonemal complex protein 2	−8.3494	4.692137
Hs.81791	NM_002546	TNFRSF11B	Tumor necrosis factor receptor superfamily, member 11b	−16.8362	0.009952
Genes not regulated by 4-HBA treatment					
Hs.431048	NM_005157	ABL1	C-abl oncogene 1, non-receptor tyrosine kinase	−1.1138	0.066919
Hs.525622	NM_005163	AKT1	V-akt murine thymoma viral oncogene homolog 1	−1.2832	0.033352
Hs.552567	NM_001160	APAF1	Apoptotic peptidase activating factor 1	−1.0289	0.640226
Hs.434980	NM_000484	APP	Amyloid beta (A4) precursor protein	1.1123	0.181838
Hs.264482	NM_004707	ATG12	ATG12 autophagy related 12 homolog (*S. cerevisiae*)	−1.0132	0.215062
Hs.529322	NM_017974	ATG16L1	ATG16 autophagy related 16-like 1 (*S. cerevisiae*)	1.0268	0.6199
Hs.477126	NM_022488	ATG3	ATG3 autophagy related 3 homolog (*S. cerevisiae*)	1.0191	0.225597
Hs.486063	NM_004849	ATG5	ATG5 autophagy related 5 homolog (*S. cerevisiae*)	−1.0137	0.143056
Hs.740389	NM_006395	ATG7	ATG7 autophagy related 7 homolog (*S. cerevisiae*)	1.0824	0.863897
Hs.624291	NM_004324	BAX	BCL2-associated X protein	1.0065	0.26068
Hs.150749	NM_000633	BCL2	B-cell CLL/lymphoma 2	1.533	0.00217
Hs.227817	NM_004049	BCL2A1	BCL2-related protein A1	−1.1823	0.709671
Hs.516966	NM_138578	BCL2L1	BCL2-like 1	−1.3254	0.177263
Hs.469658	NM_006538	BCL2L11	BCL2-like 11 (apoptosis facilitator)	3.3266	0.907015
Hs.716464	NM_003766	BECN1	Beclin 1, autophagy related	−1.1248	0.158217
Hs.696238	NM_001166	BIRC2	Baculoviral IAP repeat containing 2	1.2091	0.152718
Hs.127799	NM_001165	BIRC3	Baculoviral IAP repeat containing 3	−1.0495	0.272117
Hs.235095	NM_017891	C1orf159	Chromosome 1 open reading frame 159	1.6566	2.489837
Hs.368982	NM_032982	CASP2	Caspase 2, apoptosis-related cysteine peptidase	−1.5993	0.099699
Hs.141125	NM_004346	CASP3	Caspase 3, apoptosis-related cysteine peptidase	−1.1233	0.054198
Hs.654616	NM_032992	CASP6	Caspase 6, apoptosis-related cysteine peptidase	1.2447	0.035162
Hs.9216	NM_001227	CASP7	Caspase 7, apoptosis-related cysteine peptidase	−1.0595	0.663683
Hs.329502	NM_001229	CASP9	Caspase 9, apoptosis-related cysteine peptidase	−1.3565	0.389132
Hs.390736	NM_003879	CFLAR	CASP8 and FADD-like apoptosis regulator	1.1123	0.206097
Hs.351327	NM_017828	COMMD4	COMM domain containing 4	−1.2485	0.210456
Hs.520898	NM_001908	CTSB	Cathepsin B	−1.0859	0.166632
Hs.181301	NM_004079	CTSS	Cathepsin S	−1.5347	0.507273
Hs.578973	NM_015247	CYLD	Cylindromatosis (turban tumor syndrome)	−1.157	0.156944
Hs.654567	NM_005848	DENND4A	DENN/MADD domain containing 4A	−1.0189	0.299697
Hs.484782	NM_004401	DFFA	DNA fragmentation factor, 45 kDa, alpha polypeptide	−1.1597	0.394894
Hs.100058	NM_006426	DPYSL4	Dihydropyrimidinase-like 4	−1.5649	1.078326
Hs.158688	NM_015904	EIF5B	Eukaryotic translation initiation factor 5B	−1.4494	0.055674
Hs.667309	NM_000043	FAS	Fas (TNF receptor superfamily, member 6)	1.137	0.229523
Hs.1437	NM_000152	GAA	Glucosidase, alpha; acid	1.0603	0.628058
Hs.80409	NM_001924	GADD45A	Growth arrest and DNA-damage-inducible, alpha	1.1215	0.028813
Hs.269027	NM_014568	GALNT5	UDP-N-acetyl-alpha-D-galactosamine:polypeptide N-acetylgalactosaminyltransferase 5 (GalNAc-T5)	1.8231	0.096225
Hs.444356	NM_002086	GRB2	Growth factor receptor-bound protein 2	1.3273	0.242009
Hs.29169	NM_024610	HSPBAP1	HSPB (heat shock 27 kDa) associated protein 1	1.3892	1.706257
Hs.643120	NM_000875	IGF1R	Insulin-like growth factor 1 receptor	−1.1904	0.05958
Hs.632273	NM_181509	MAP1LC3A	Microtubule-associated protein 1 light chain 3 alpha	−1.0059	0.049965
Hs.138211	NM_002750	MAPK8	Mitogen-activated protein kinase 8	1.0652	0.595847
Hs.632486	NM_021960	MCL1	Myeloid cell leukemia sequence 1 (BCL2-related)	1.1497	0.049134
Hs.618430	NM_003998	NFKB1	Nuclear factor of kappa light polypeptide gene enhancer in B-cells 1	−1.0531	0.547016
Hs.513667	NM_003946	NOL3	Nucleolar protein 3 (apoptosis repressor with CARD domain)	−1.7116	0.206745
Hs.177766	NM_001618	PARP1	Poly (ADP-ribose) polymerase 1	−1.0047	0.048371
Hs.409412	NM_005484	PARP2	Poly (ADP-ribose) polymerase 2	1.0899	0.160191
Hs.171844	NM_006505	PVR	Poliovirus receptor	−1.0185	0.2427
Hs.463642	NM_003161	RPS6KB1	Ribosomal protein S6 kinase, 70 kDa, polypeptide 1	1.1352	0.310545
Hs.21374	NM_000345	SNCA	Synuclein, alpha (non A4 component of amyloid precursor)	−1.7882	0.009952
Hs.48513	NM_006038	SPATA2	Spermatogenesis associated 2	−1.3653	0.694708
Hs.587290	NM_003900	SQSTM1	Sequestosome 1	−1.073	0.076573
Hs.189782	NM_018202	TMEM57	Transmembrane protein 57	−1.0805	0.149693
Hs.241570	NM_000594	TNF	Tumor necrosis factor	−1.7491	0.009952
Hs.591834	NM_003844	TNFRSF10A	Tumor necrosis factor receptor superfamily, member 10a	1.2277	0.200162
Hs.713833	NM_001065	TNFRSF1A	Tumor necrosis factor receptor superfamily, member 1A	−1.4144	0.047858
Hs.437460	NM_000546	TP53	Tumor protein p53	−1.1421	0.259099
Hs.522506	NM_021138	TRAF2	TNF receptor-associated factor 2	−1.4742	0.036574
Hs.134406	NM_017853	TXNL4B	Thioredoxin-like 4B	−1.2299	0.083412
Hs.47061	NM_003565	ULK1	Unc-51-like kinase 1 (C. elegans)	−1.7787	0.146981

Regarding other death cell signaling pathways^19^, the observed up-regulation of the gene encoding X-linked inhibitor of apoptosis (XIAP) indicated that the 4-HBA treatment inhibited the canonical apoptotic pathway in A549 cells. Furthermore, microarray results highlighted that genes involved in the extrinsic apoptotic pathway were strongly down-regulated: CD40 ligand (CD40L), Fas ligand (FasL), Myelin associated glycoprotein (MCL1) and Tumor necrosis factor receptor superfamily, member 11b (TNFRS11B). As for the expression of key genes involved in the necrotic pathway, they were also down-regulated: Forkhead box I1 (FOXL1), Olfactory receptor, family 10, subfamily J, member 3 (OR10J3), S100 calcium binding protein A7A (S100A7A) and Junctophilin 3 (JPH3). Finally, homeostatic and detoxification cell pathways such as autophagy were also strongly down-regulated: Interferon gamma (IFNG), Estrogen receptor 1 (ESR1), Huntingtin (HTT), Insulin-like growth factor 1 (IGF1), Insulin (INS), Immunity-related GTPase family M (IRGM), Kv channel interacting protein 1 (KCNIP1), RAB25 member RAS oncogene family and Synaptonemal complex protein 2 (SYCP2). The genes involved in other death cell signaling pathways were not differently expressed in A549 cells treated with 4-HBA (Table 1).

Since caspase-1 plays a crucial role in the pyroptotic cell signaling pathway, its protein levels in A549 treated cells were analyzed by immunoblot (Figure S5). 4-HBA treatment was carried out under the same conditions used for PCR array analysis (1 µg ml^−1^), but the cells were collected after 24 hours, since changes in protein levels were more evident at this time point. In Fig. 3, the increase in pro-caspase-1 levels (panel B) and appearance of the P20 caspase-1 protein cleavage fragment (panel C) can be seen only in treated samples, thus confirming that A549 cell death induced by 4-HBA treatment was due to caspase-1 activation.

## Discussion

Marine organisms represent an attractive source of marine natural products in view of the high hit rates of marine molecules as antitumor and antibiotic drug leads^21^. Especially, polar marine bacteria are an unexploited hoard of biodiversity equipped with an interesting chemical repertoire^13^. In this study, we evaluated the antiproliferative activity on tumor cells of ethyl acetate crude extracts of cold-adapted bacteria belonging to the genera *Pseudoalteromonas*, *Pseudomonas*, *Psychrobacter* and *Psychromonas*. Even though nine out of the thirteen analysed strains belong to the same genus (*Pseudoalteromonas*), their recent genome-wide comparison attested a quite remarkable diversity, with a large proportion of unique genes^22^. This analysis prompted us to use all thirteen strains in the initial screening. The *P. haloplanktis* TAC125 crude extract was shown to be the most active in inhibiting cell proliferation. The bioactivity guided purification scheme highlighted the presence of more than one fraction endowed with anti-proliferative activity when tested on A549 cells. The fraction that was non toxic on WI-38 cells was subjected to a further fractionation step. 4-Hydroxybenzoic acid (4-HBA) was identified as the single compound responsible for the observed antiproliferative activity. 4-HBA is a primary metabolite as it is one of the products of chorismate lyase, which converts chorismic acid into pyruvate and 4-HBA. The latter molecule is then addressed towards the ubiquinone biosynthetic pathway. It is interesting to note that another *Pseudoalteromonas* strain*, Pseudoalteromonas flavipulchra*, was reported to produce 4-HBA, to which an antimicrobic activity was assigned^23^. In this paper, we demonstrate that 4-HBA inhibits A549 cancer cell proliferation with an IC_50_ value ≤1 μg ml^−1^. Although we have not currently identified the cellular target(s) of 4-HBA, we show that its effect is specific, since the other HBA isomers (i.e. 2-HBA and 3-HBA) were unable to induce any anti-proliferative effect on the cancer cell line tested. Furthermore, the effect of 4-HBA is selective, as the treatment of normal lung epithelium cell line (WI-38) with 4-HBA does not affect cell viability.

We demonstrate that at gene and protein levels, 4-HBA is able to activate pyroptosis in A549 cells. Pyroptotic cell death was reported as a caspase-1-dependent cell death of macrophages when infected by *Salmonella* typhimurium^24,25^. In contrast to apoptosis, pyroptosis is a pro-inflammatory type of cell death due to the activation of caspase-1 leading to the formation of the inflammasome, resulting in the release of the pro-inflammatory cytokines IL-1β and IL-18^3^. Our results show that treatment of A549 cancer cells with 4-HBA induces the transcription of (amongst others) caspase-1, IL1β, and IL18 encoding genes. Furthermore, a clear accumulation of caspase-1 and its activated form (pro-caspase-1) was observed at the protein level. Interestingly, the only death signaling pathway activated was pyroptosis, as demonstrated by the down-regulation of key genes involved in apoptosis, necrosis and autophagy. This clearly indicates that 4-HBA is specifically recognized by tumor cells that fall into the inflammasome cascade. At the morphological level, 4-HBA treated cells showed a typical membrane swelling ascribable to cell lysis occurring during pyroptosis. The observation that the viability of WI-38 was not affected by treatment with 4-HBA suggests that this microbial derived metabolite cannot be considered a bacterial virulence factor. On the other hand, its selective anti-proliferative activity against cancer cells (at least the tested in the present study) indicates a molecular target mainly present in transformed cells. To test if 4-HBA was also able to inhibit the proliferation of other transformed cell lines, colon rectal adenocarcinoma (HT29) cell line was selected and subjected to treatment with all HBA chemical isomers (Figure S6). Again only 4-HBA was able to inhibit HT29 cell proliferation.

Data reported here disclose the role of 4-HBA as a novel inducer of pyroptosis. We forecast that 4-HBA will become a useful tool for elucidating still unknown molecular relationships between pyroptosis and cancer.

## Methods

### Bacterial strains and culture conditions

Each Antarctic bacteria strain (see Table S1) were grown aerobically in flasks at 15 °C in GG defined medium^16^ (10 g L^−1^ L-Glutamate, 10 g L^−1^ D-Gluconate, 1 g L^−1^ K_2_HPO_4_, 10 g L^−1^ NaCl, 1 g L^−1^ NH_4_NO_3_, 200 mg L^−1^ MgSO_4_∙7H_2_O, 5 mg L^−1^ FeSO_4_∙7H_2_O, 5 mg L^−1^ CaCl_2_∙2H_2_O pH 7.5) or TYP medium^17^ (16 g L^−1^ yeast extract, 16 g L^−1^ bacto Tryptone, 10 g L^−1^ NaCl). At the end of the exponential phase cells and spent medium were harvested.

### Bacterial fermentation

*Pseudoalteromonas haloplanktis* TAC125 growth was performed in a Stirrer Tank Reactor 7L Bioreactor Techfors S (INFORS HT) connected to an IRIS 5.0 software with a working volume of 7L. Fermentation was performed in 5L of GG defined medium. The bioreactor was equipped with the standard pH, pO_2_, and temperature sensor for bioprocess monitoring. The culture was carried out at 15 °C for 31 hours in aerobic conditions DOT (dissolved oxygen tension) ≥20%, using an airflow of 1.5 L/hour, and a stirrer speed of 250 rpm.

### Total bacterial extracts preparation

Cold-adapted bacterial cultures (cells and spent medium), previously frozen at −80 °C, without adding cryoprotectants, were thawed and stirred with an equal volume of ethyl acetate (Assay Percent Range ≥99.5%) (Sigma-Aldrich) and mixed with 1% formic acid (Assay Percent Range = 90%) (JT Baker). Each solution was stirred at least for 30 min and then centrifuged at 3000 rpm for 30 min. The resulting two phases were separated and the organic phases were recovered and dried using a rotary evaporator, Rotavapor (Buchi R-210) at 40 °C. The resulting total organic extracts were dispensed and stored at −20 °C.

### Anticancer compound purification and identification

#### Sample purification

The crude extract was pre-fractionated on a reversed phase C_18_ flash column (10 g, 15 ml) using an Isolera One automated flash system (BIOTAGE, Uppsala, Sweden). The gradient was 10% stepwise (15 column volumes) from 30–100% methanol (MeOH) buffered with 20 mM formic acid with a flow of 15 ml/min. Nine fractions were collected manually every 10% step. MeOH was of HPLC grade and water was purified and deionized using a Millipore system through a 0.22 μM membrane filter (Milli-Q water).

4-Hydroxybenzoic acid (fraction 1E) was purified from the Isolera fraction (fraction 1) on a Waters semi-preparative HPLC, with a Waters 600 Controller (Milford, MA, USA) coupled to a Waters 996 Photodiode Array Detector. Separation was achieved on a Luna II C18, 5 μm, 250 × 10 mm column (Phenomenex, Torrance, CA, USA) with a flow of 5 ml min^−1^ using a linear gradient 5% MeCN in Milli-Q water with 50 ppm TFA going to 35% MeCN in 24 min, from 35–45% MeCN in 2 min, 45–100% MeCN in 2 min, kept for 5 min at 100% MeCN and down to the starting conditions in 2 min. MeCN was of HPLC grade.

#### 4-HBA identification

The identification of 4-HBA was performed using ultra-high performance liquid chromatography-diode array detection-quadrupole time of flight mass spectrometry (UHPLC-DAD-QTOFMS) with tandem HRMS fragmentation on an Agilent Infinity 1290 UHPLC system (Agilent Technologies, Santa Clara, CA, USA) equipped with a DAD and an Agilent 6550 iFunnel QTOF MS (as previously described^18^) and comparing results obtained with spectra acquired using the commercial standards.

#### Commercial standards

4-hydroxybenzoic acid, 3-hydroxybenzoic acid and 2-hydroxybenzoic acid (salicylic acid) were purchased at Sigma-Aldrich (Steinheim, Germany).

#### Treatment of Human Cells

The adenocarcinoma human alveolar basal epithelial cell line A549 was purchased from the American Type Culture Collection (ATCC^®^ CCL185™) and grown in DMEM-F12 (Dulbecco’s modified Eagle’s medium) supplemented with 10% (v/v) of fetal bovine serum (FBS), 100 units ml^−1^ penicillin and 100 µg ml^−1^ streptomycin. The normal diploid human lung fibroblasts WI-38 were purchased from the American Type Culture Collection (ATCC^®^ CCL-75™) and grown in MEM supplemented with 10% (v/v) of fetal bovine serum (FBS), 100 units ml^−1^ penicillin and 100 µg ml^−1^ streptomycin, 2 mM of L-glutamine and non-essential amino acids (NEAA, 2 mM). The human colorectal adenocarcinoma cell line HT29 was purchased from the American Type Culture Collection (ATCC® HTB38) and maintained in McCoy’s 5 A medium supplemented with 10% (v/v) fetal bovine serum (FBS), 2 mmole L^−1^ glutamine and 100 units ml^−1^ penicillin and 100 µg ml^−1^ streptomycin. The medium was renewed every 3 days, and the cells were detached via trypsinization when they reached confluence. Before the experiments, cells were seeded in 96-well plates (2 × 10^3^ cells well^−1^) and kept overnight for attachment. For viability assays, the extracts, fractions and pure compound(s) were dissolved in dimethyl sulfoxide at a final concentration of 1% (v/v) for each treatment.

#### MTT- Viability assay

The effect of extracts, fractions and pure compound(s) on cell viability were determined using the 3-(4,5-Dimethylthiazol-2-yl)-2,5-Diphenyltetrazolium Bromide (MTT) assay (Applichem A2231). A549, HT29 and WI-38 cells, seeded in 96-well plates, after treatment times (24 and 48 hours), were treated with 10 µl (5 mg ml^−1^) of MTT and incubated for 3 hours. After the incubation time, isopropanol was used to dissolve purple formazan crystals. The absorbance was recorded on a microplate reader at a wavelength of 570 nm (Multiskan FC, THERMO SCIENTIFIC). The effect on cell viability was evaluated as percent of cell viability calculated as the ratio between mean absorbance of each sample and mean absorbance of controls.

### RT^2^ profiler PCR-array analysis for cell death pathway identification

A549 (2 × 10^6^) cells used for RNA extraction and analysis, were seeded in Petri dishes (100 mm diameter) to obtain the control condition without any treatment and cells treated with the IC_50_ concentration of the pure compound (1 µg ml^−1^). After 2 hours of exposure time, A549 cells were washed directly in the Petri dish by adding cold Phosphate-Buffered Saline (PBS) and rocking gently.

Cells were lysed in the Petri dish by adding 1 ml of Trisure Reagent (Bioline, cat. BIO-38033) per 100 mm dish diameter. RNA was isolated according to the manufacturer’s protocol. RNA concentration and purity was assessed using the nanophotomer NanodroP (Euroclone).

About 200 ng RNA was subjected to reverse transcription reaction using the RT^2^ first strand kit (Qiagen, cat. 330401) according to the manufacturer’s instructions. The qRT-PCR analysis was performed in triplicate using the RT^2^ Profiler PCR Array kit (Qiagen, cat. 330231), in order to analyze the expression of death cell signaling genes on A549 cells. Plates were run on a ViiA7 (Applied Biosystems 384 well blocks), Standard Fast PCR Cycling protocol with 10 µl reaction volumes. Cycling conditions used were: 1 cycle initiation at 95.0 °C for 10 min followed by amplification for 40 cycles at 95.0 °C for 15 s and 60.0 °C for 1 min. Amplification data were collected via ViiA 7 RUO Software (Applied Biosystems). The cycle threshold (Ct)-values were analyzed with PCR array data analysis online software (http://pcrdataanalysis.sabiosciences.com/pcr/arrayanalysis.php, Qiagen). Control genes for Real-Time qPCR were actin-beta (ACTB), beta-2-microglobulin (B2M), hypoxanthine phosphoribosyltransferase (HPRT1) and ribosomal protein, large subunit P0 (RPLP0), the expression of which remained constant in A549 cells.

### Protein extraction and immunoblot analysis

A549 cells (2 × 10^6^), were seeded in Petri dishes (100 mm diameter) and cultured without any treatments (untreated control) or with the IC_50_ concentration of the pure compound (4-HBA, 1 µg ml^−1^). A549 cell lysates were prepared after 24 hours of treatment by scraping the cells of each Petri dish into 1 ml of Radio Immune Precipitation Assay buffer (RIPA, Cell Signaling, cat. 9806), supplemented with 1 µM of protease inhibitor PMSF (Cell Signaling, cat. 8553). The lysates were incubated on ice for 15 min and then clarified by centrifugation at 14000 × *g*, for 20 min. Total protein concentrations were determined according to the Bradford method using the Protein Assay Reagent (Applichem, cat. A6932) with bovine serum albumin (BSA, Sigma Aldrich, cat. A2058) as standard. The protein extracts were stored at −20 °C until use. Before electrophoresis, protein samples were incubated at 100 °C for 5 min. Following 10% SDS-PAGE, gels were stained with Comassie or blotted onto nitrocellulose membrane (Biorad, cat. 170–4159). Membranes were incubated for 1 hour in blocking reagent (1 × Tris Buffered Saline-TBS), with 0.1% Tween-20 with 5% w/v nonfat dry milk, and incubated overnight at 4 °C with the primary antibodies diluted in 1 × TBS, 0.1% Tween-20 with 5% BSA (CASP1, 1:1000, Biorbyt orb10232).

After incubation, membranes were washed three times for 10 min each with 15 ml of TBS/Tween and then incubated with HRP-conjugated secondary antibody with gentle agitation for 1 h at room temperature. For β-actin, we used HRP-conjugated secondary antibody anti-mouse (1:10000, Santa Cruz Biotechnology); for CASP1 we used HRP-conjugated secondary antibody anti-rabbit (1:10000, Jackson ImmunoResearch).

After incubation, membranes were washed three times for 10 min each with 15 ml of TBS/Tween. Blotted membranes were immunodetected using clarity Western ECL (Biorad, cat. 170-5060). Proteins were visualized with Fuji medical X-ray film (cat. 47410). Densitometric analysis of immunopositive bands was performed using ImageJ software.

## Electronic supplementary material

Supporting file
