# Supplementary material for: Pseudoalteromonas haloplanktis TAC125 produces 4-hydroxybenzoic acid that induces pyroptosis in human A459 lung adenocarcinoma cells
Source: Sci Rep. 2018 Jan 19;8:1190. doi: 10.1038/s41598-018-19536-2 (PMC5775203; doi:10.1038/s41598-018-19536-2)
Supplement: Supplementary file 1 — Supporting file [file 41598_2018_19536_MOESM1_ESM.doc]

***Pseudoalteromonas haloplanktis* TAC125 produces 4-hydroxybenzoic acid that induces pyroptosis in human A459 lung adenocarcinoma cells.**

Filomena Sannino^1,*^, Clementina Sansone^2^,*, Christian Galasso^2^, Sara Kildgaard^3^, Pietro Tedesco^4^, Renato Fani^5^, Gennaro Marino^1,6^, Donatella de Pascale^4^, Adrianna Ianora^2^, Ermenegilda Parrilli^1^, Thomas Ostenfeld Larsen^3^, Giovanna Romano^2,§^, Maria Luisa Tutino^1,§^

^1^Department of Chemical Sciences, University of Naples "Federico II", Complesso Universitario Monte S. Angelo, Via Cintia, Naples 80126, Italy.

^2^Integrative Marine Ecology Department, Stazione Zoologica Anton Dohrn, Villa Comunale, Naples 80121, Italy.

^3^Department of Biotechnology and Biomedicine, Søltofts Plads, Building 221, Technical University of Denmark, DK-2800 Kgs. Lyngby, Denmark.

^4^Institute of Protein Biochemistry, CNR, Via Pietro Castellino 111, Naples 80131, Italy.

^5^Department of Biology, LEMM, Laboratory of Microbial and Molecular Evolution Florence, University of Florence, I-50019, Sesto Fiorentino (FI), Italy.

^6^University Suor Orsola Benincasa, Via Santa Caterina da Siena, 32, Naples 80132, Italy.

*These authors equally contributed to the work

^§^ Corresponding authors romano@szn.it, tutino@unina.it

**Table S1: Antarctic bacterial strains used in this work**

| Strain | Origin | Reference and/or source |
| --- | --- | --- |
| *Pseudomonas sp.* TAA207 | Antarctic sea water | Presta L. et al, 2016, Genome Announc. Jul 28;4(4) |
| *Pseudomonas sp.* TAD18 | Antarctic sea water | Presta L. et al, 2016, Genome Announc. Jul 28;4(4) |
| *P. haloplanktis* TAE56 | Antarctic sea water | Bosi et al. 2015- Hydrobiologia, 761:85-95 |
| *P. haloplanktis* TAE57 | Antarctic sea water | Bosi et al. 2015, Hydrobiologia, 761:85-95 |
| *P. haloplanktis* TAE80 | Antarctic sea water | Bosi et al. 2015, Hydrobiologia, 761:85-95 |
| *P. haloplanktis* TAB23 | Antarctic sea water | Feller G. *et al* 1992, J. Biol. Chem. 267:5217–5221 |
| *P. haloplanktis* TAC125 | Antarctic sea water | Médigue *et al.*, 2005, Genome Res. 15:1325-35 |
| *P. haloplanktis* TB13 | Antarctic sea water | Bosi et al. 2015, Hydrobiologia, 761:85-95 |
| *P. haloplanktis* TB51 | Antarctic sea water | Bosi et al. 2015, Hydrobiologia, 761:85-95 |
| *P. haloplanktis* TB64 | Antarctic sea water | Bosi et al. 2015, Hydrobiologia, 761:85-95 |
| *Psychromonas arctica* | Arctic sea water | Groudieva et al., 2003, Int J Syst Evol Microbiol. 53:539-45 |
| *Psychrobacter sp.* TAD1 | Antarctic sea water | Di Fraia et al. 2000, Eur. J. Biochemistry 267:121-131 |
| *Pseudoalteromonas sp.* TB41 | Antarctic sea water | Bosi et al. 2015, Hydrobiologia, 761:85-95 |

Supplementary figure S1


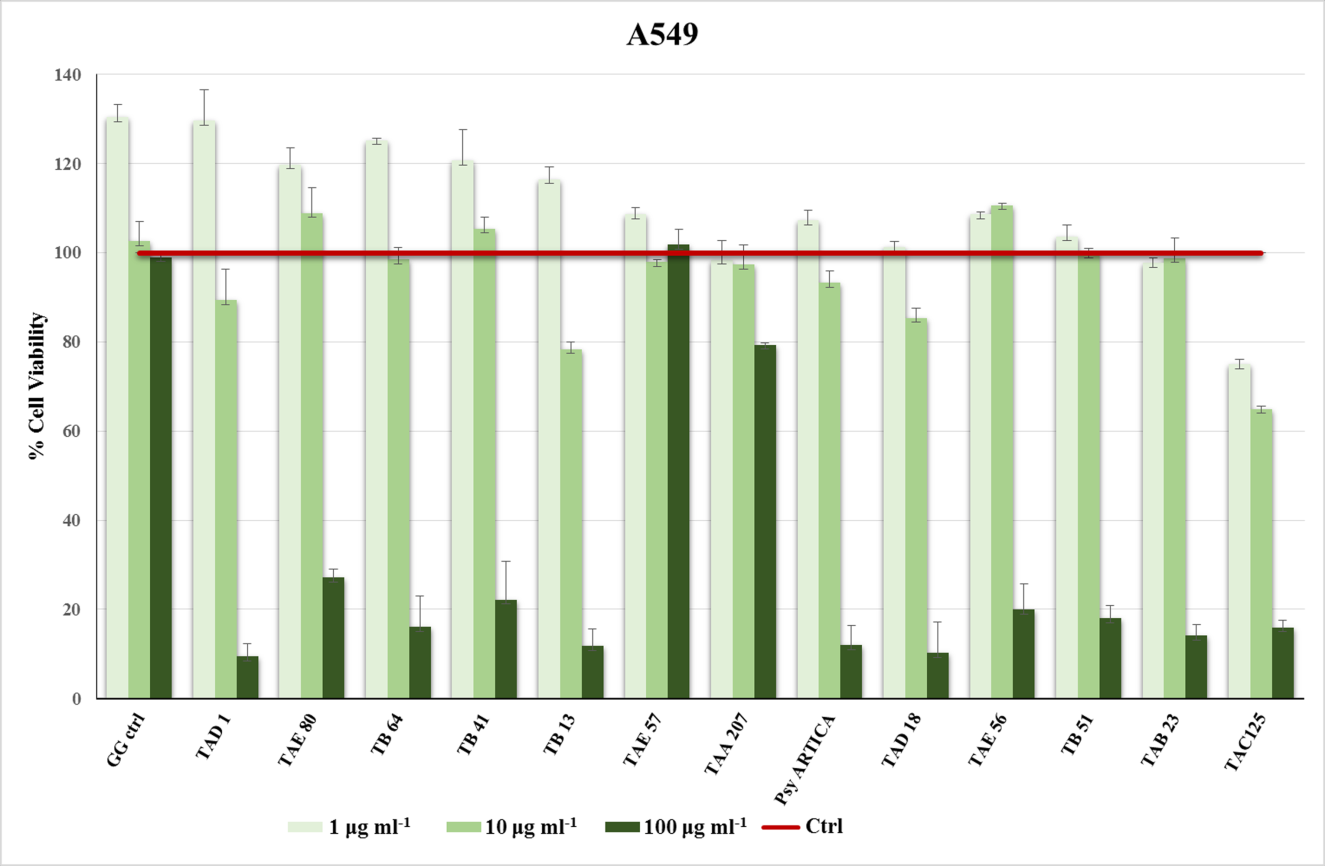


**FIGURE S1:** Cell viability of lung adenocarcinoma cells (A549) treated for 48 hours with three different concentrations (1, 10 and 100 µg ml^-1^) of crude extracts from 13 cold-adapted bacteria. Red bar represents untreated cells (control).

Three independent assays were performed in triplicate; viability data are shown as mean ± S.D.

Supplementary figure S2


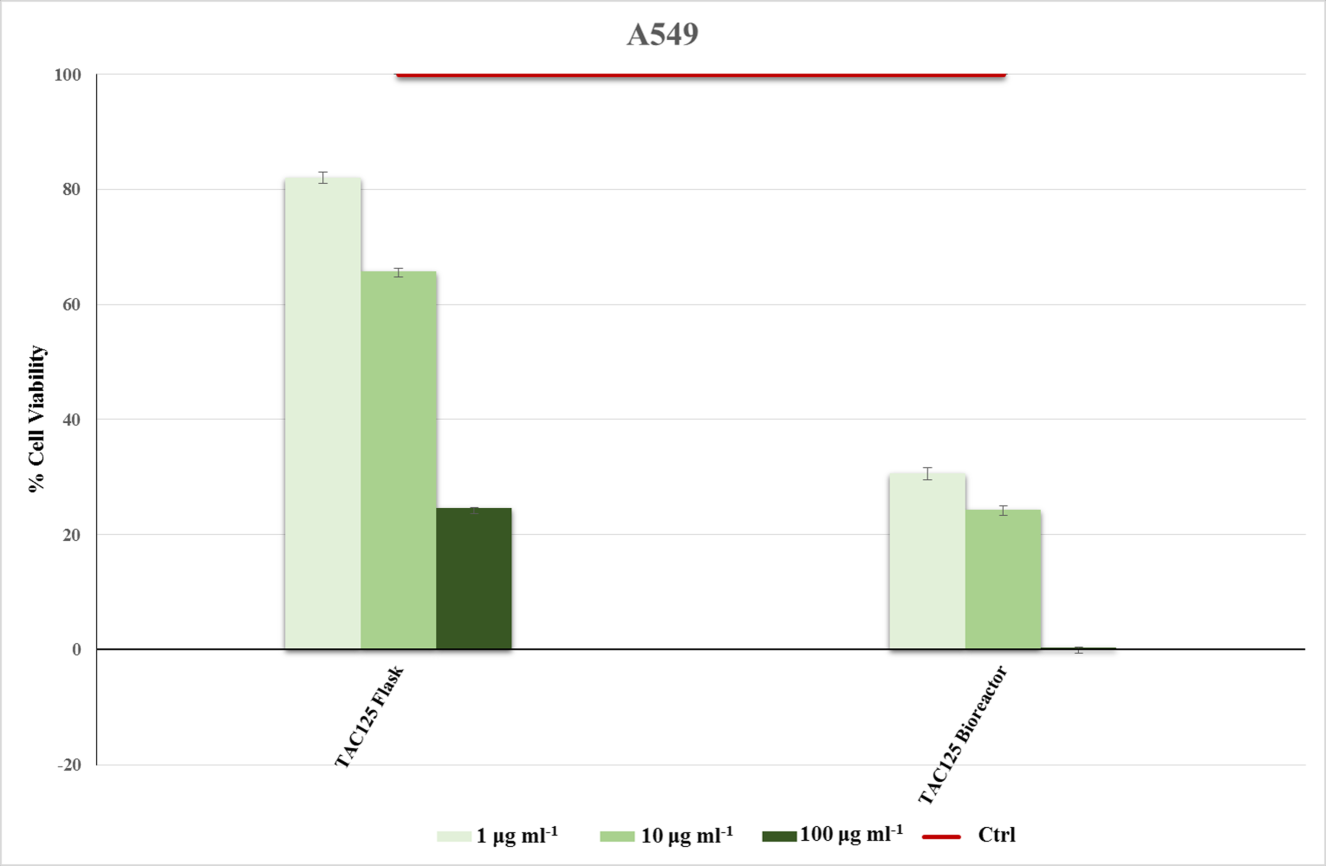


**FIGURE S2:** Cell viability of lung adenocarcinoma cells (A549) treated for 48 hours with three different concentrations (1, 10 and 100 µg ml^-1^) of two crude extracts of *Pseudoalteromonas haloplanktis* TAC125 grown in different laboratory systems: flask and bioreactor. Red bar represents untreated cells (control). Three independent assays were performed in triplicate; viability data are shown as mean ± S.D.

Supplementary figure S3


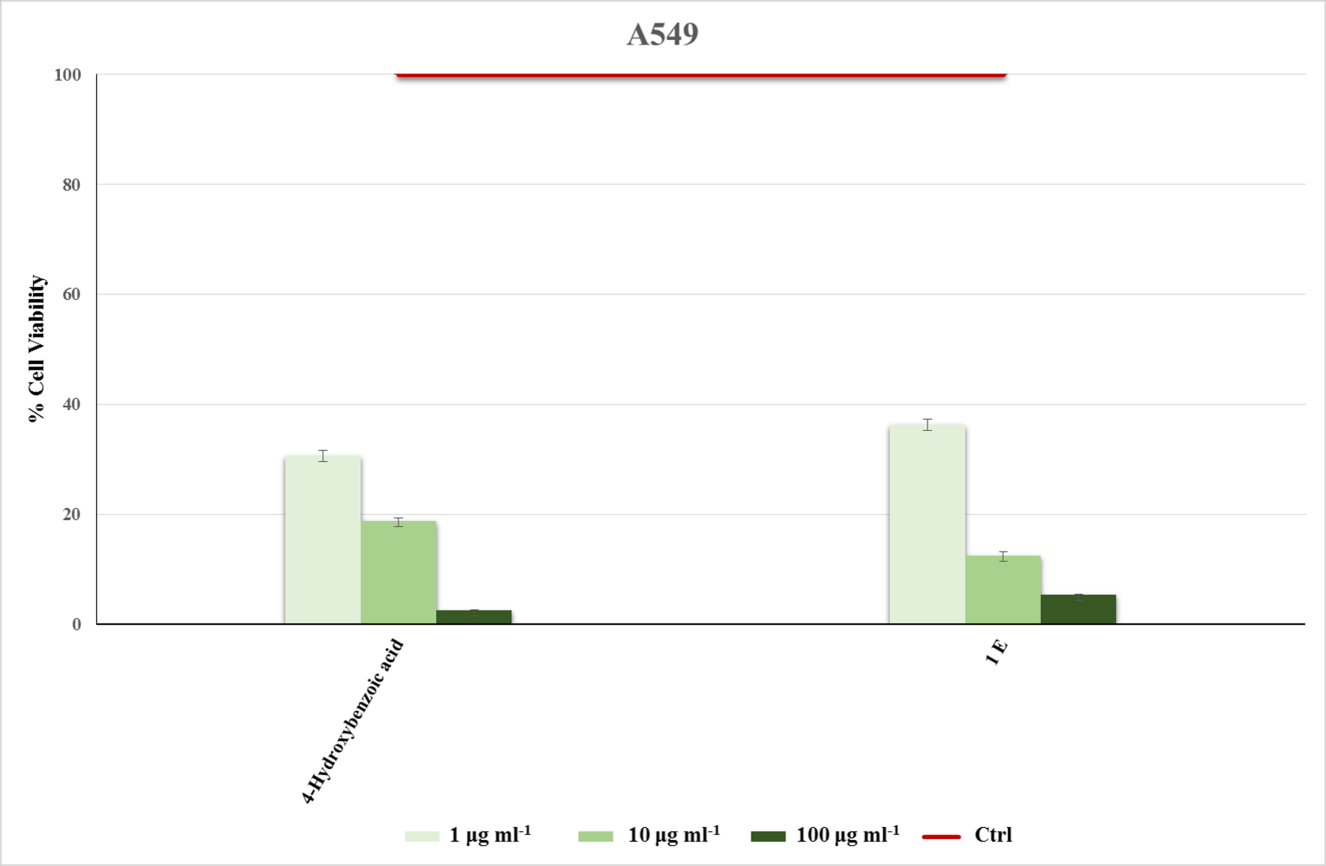


**FIGURE S3:** Cell viability of lung adenocarcinoma cells (A549) treated for 48 hours with three different concentrations (1, 10 and 100 µg ml^-1^) of two samples: commercial 4-hydroxybenzoic acid and purified 4-hydroxybenzoic acid (1E). Red bar represents untreated cells (control). Three independent assays were performed in triplicate; viability data are shown as mean ± S.D.

Supplementary figure S4


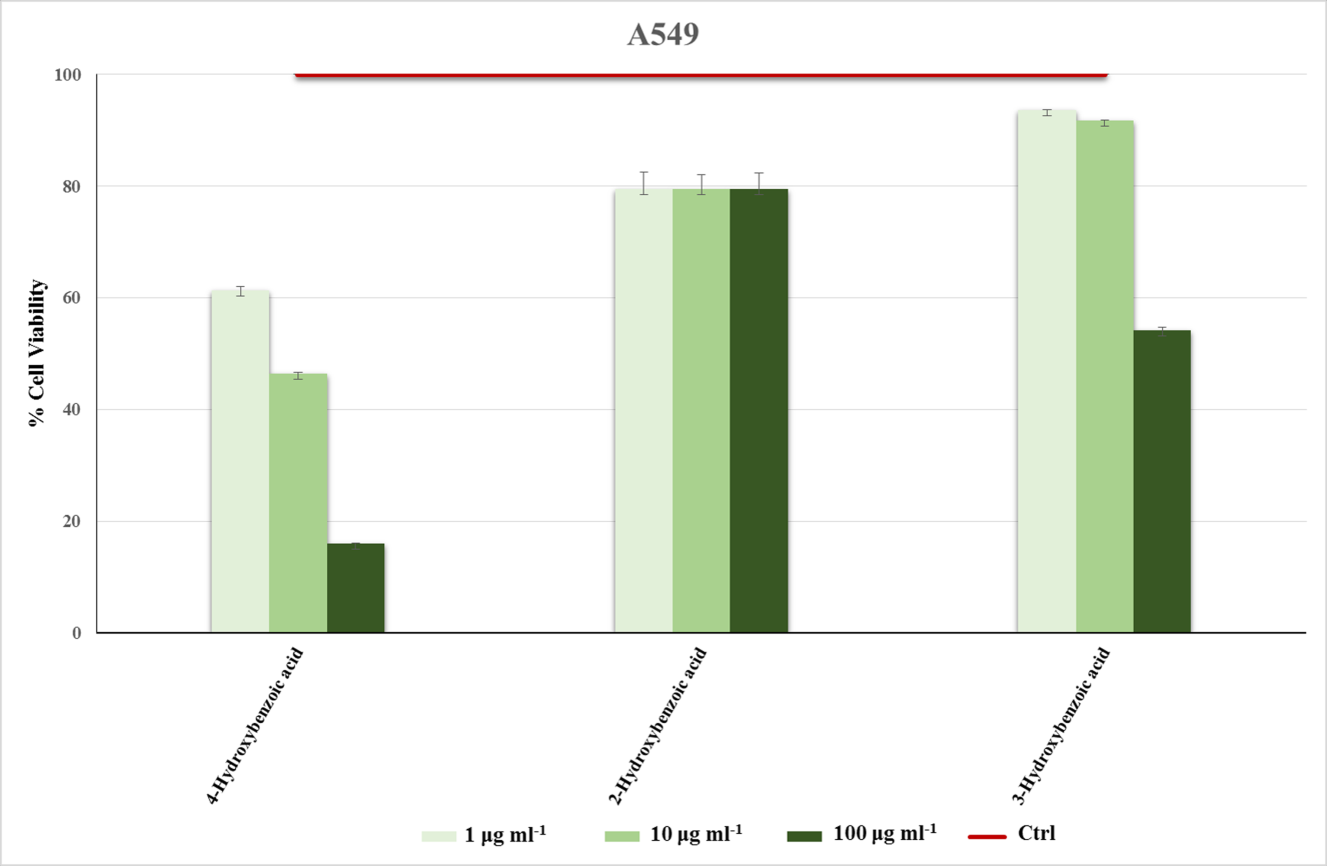


**FIGURE S4**: Cell viability of lung adenocarcinoma cells (A549) treated for 48 hours with three different concentrations (1, 10 and 100 µg ml^-1^) of the three structural isomers (4-, 2- and 3-Hydroxybenzoic acid). Red bar represents untreated cells (control).

Three independent assays were performed in triplicate; viability data are shown as mean ± S.D.

Supplementary figure S5


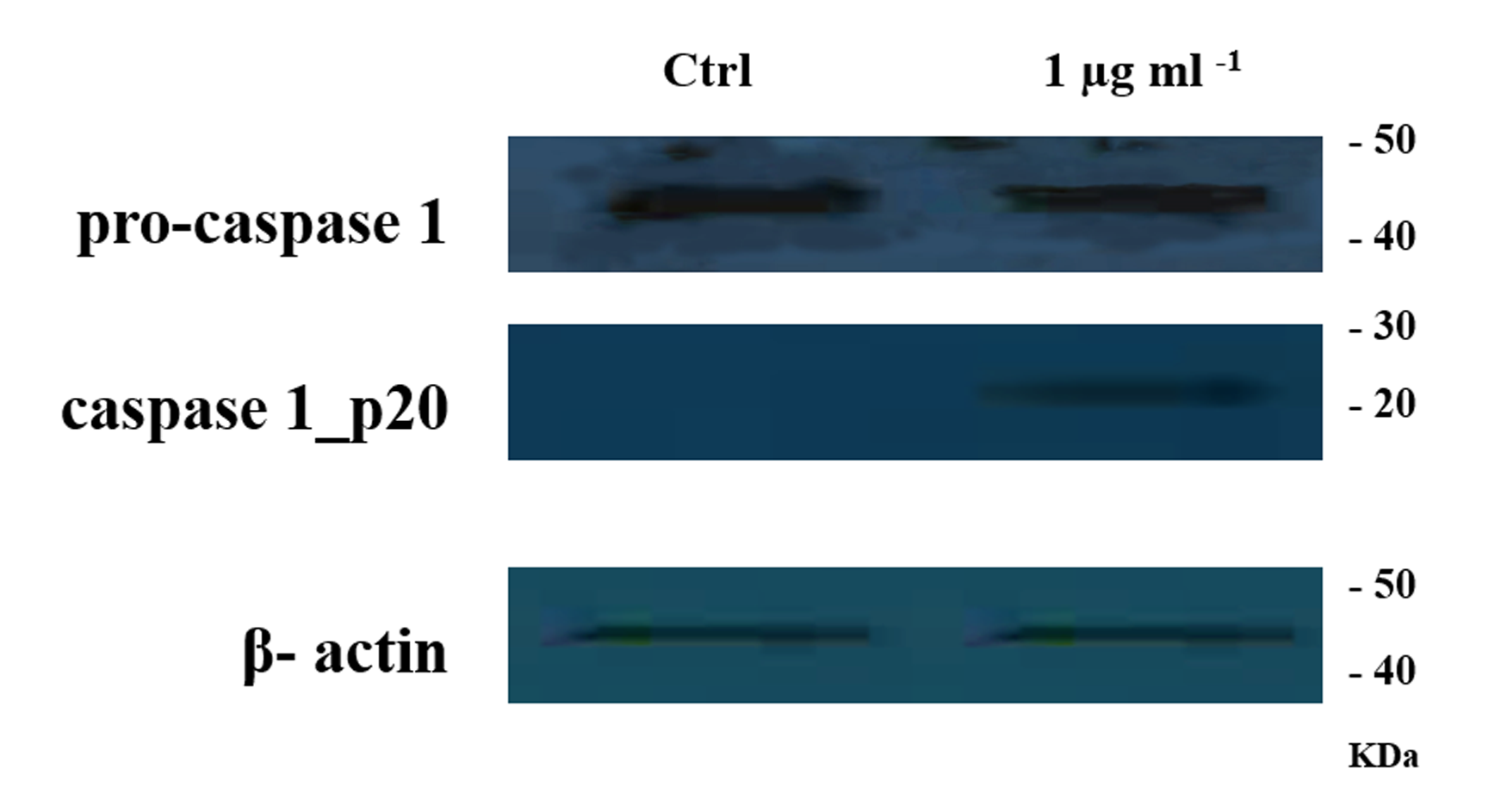


**FIGURE S5**:Three independent assays were performed in triplicate on A549 cells treated with 1 μg ml^-1^ and variation in protein expression was obtained by immunoblot analysis. The image represents photographic sheets measuring the variation in protein expression.

Supplementary figure S6


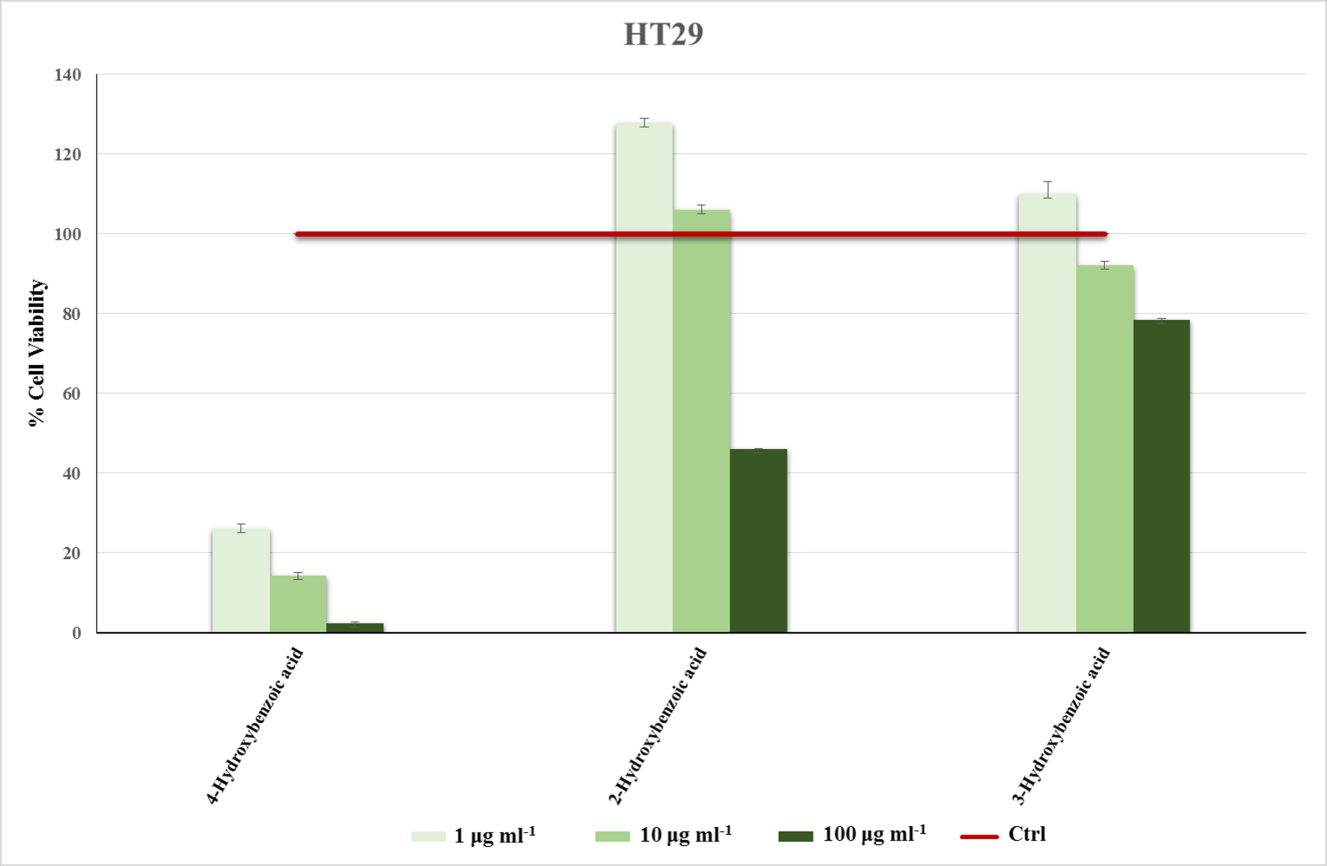


**Figure S6:** Cell viability of colon rectal adenocarcinoma cells (HT29) treated for 48 hours with three different concentrations (1, 10 and 100 µg ml^-1^) of the three structural isomers: 4-, 2- and 3-hydroxybenzoic acid. Red bar represents untreated cells (control). Three independent assays were performed in triplicate; viability data are shown as mean ± S.D.
